# The first laminin G-like domain of protein S is essential for binding and activation of Tyro3 receptor and intracellular signalling

**DOI:** 10.1016/j.bbrep.2022.101263

**Published:** 2022-04-28

**Authors:** Nour Al Kafri, Josefin Ahnström, Adrienn Teraz-Orosz, Ludovic Chaput, Natesh Singh, Bruno O. Villoutreix, Sassan Hafizi

**Affiliations:** a School of Pharmacy and Biomedical Sciences, University of Portsmouth, Portsmouth, UK; b Faculty of Medicine, Dept. of Immunology and Inflammation, Imperial College London, UK; c University of Lille, Inserm, Institut Pasteur de Lille, U1177 - Drugs and Molecules for Living Systems, F-59000, Lille, France

**Keywords:** Signal transduction, Protein-protein interaction, Receptor tyrosine kinase, Homology modelling, Molecular docking, Protein structure, Tyro3, Gas6, Protein S, Laminin G domain, Ligand-receptor binding

## Abstract

The homologous proteins Gas6 and protein S (ProS1) are both natural ligands for the TAM (Tyro3, Axl, MerTK) receptor tyrosine kinases. ProS1 selectively activates Tyro3; however, the precise molecular interface of the ProS1-Tyro3 contact has not been characterised. We used a set of chimeric proteins in which each of the *C*-terminal laminin G-like (LG) domains of ProS1 were swapped with those of Gas6, as well as a set of ProS1 mutants with novel added glycosylations within LG1. Alongside wildtype ProS1, only the chimera containing ProS1 LG1 domain stimulated Tyro3 and Erk phosphorylation in human cancer cells, as determined by Western blot. In contrast, Gas6 and chimeras containing minimally the Gas6 LG1 domain stimulated Axl and Akt phosphorylation. We performed *in silico* homology modelling and molecular docking analysis to construct and evaluate structural models of both ProS1-Tyro3 and Gas6-Axl ligand-receptor interactions. These analyses revealed a contact between the ProS1 LG1 domain and the first immunoglobulin domain of Tyro3, which was similar to the Gas6-Axl interaction, and involved long-range electrostatic interactions that were further stabilised by hydrophobic and polar contacts. The mutant ProS1 proteins, which had added glycosylations within LG1 but which were all outside of the modelled contact region, all activated Tyro3 in cells with no hindrance. In conclusion, we show that the LG1 domain of ProS1 is necessary for activation of the Tyro3 receptor, involving protein-protein interaction interfaces that are homologous to those of the Gas6-Axl interaction.

## Highlights

• The first LG domain (LG1) of protein S is essential for activation of Tyro3 and downstream signalling in human cancer cells.
• The LG1 domain is necessary but not sufficient for TAM activation, as the presence of a tandem LG2 domain is also required.
• There is a similarity between the Gas6-Axl and ProS1-Tyro3 contact sites.

## 1 Introduction

**Fig. 1:**
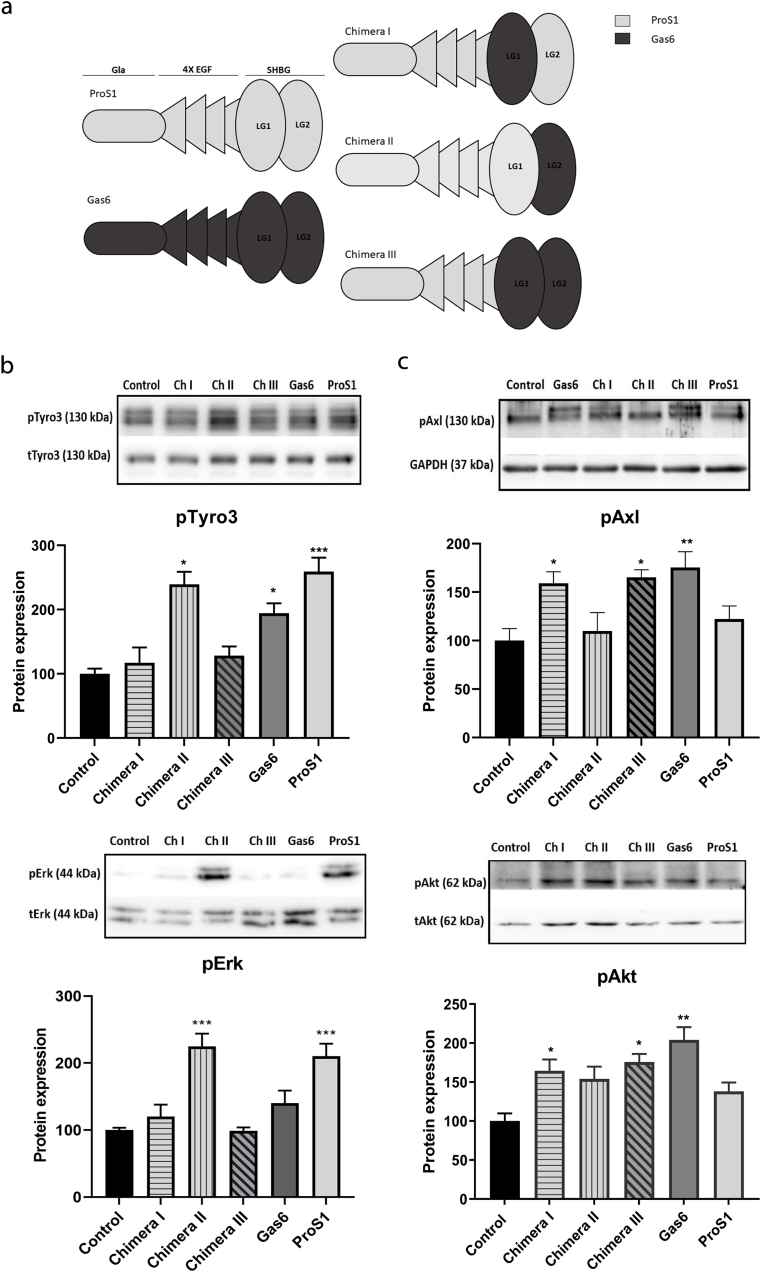
The effects of ProS1, Gas6 and chimeras on TAM receptor and coupled downstream signalling molecule activation in SCC-25 cells. (a) Schematic representation of recombinant TAM ligand constructs used in this study. These included human ProS1, Gas6, and three ProS1/Gas6 chimeras. All of the chimeras contained the Gla domain and EGF-like domains of ProS1. Light grey colour denotes regions corresponding to ProS1 amino acid sequence, whereas dark grey denotes regions corresponding to Gas6 amino acid sequence. (b) Western blot showing phosphorylated Tyro3 (pTyro3) and Erk (pErk) levels after stimulation with recombinant Gas6, ProS1 and three chimeras (7.5 nM) for 9 min. (c) Western blot showing phosphorylated Axl (pAxl) and Akt (pAkt) levels under the same experimental conditions as in (b). Each representative blot image is followed by accompanying graphs of densitometric quantification of bands (n = 3 separate experiments). Data as mean ± SEM expression for each phosphoprotein was normalized against the total protein/loading control (tTyro3, tERK, GAPDH, actin). ANOVA with Tukey's multiple comparison *post-hoc* analysis; ***p < 0.001, **p < 0.01, *p < 0.05 versus control (untreated). While the sample loading order is different in the pAxl blot, the quantification bar charts are presented in the same order for consistency.

The homologous vitamin K-dependent proteins growth arrest specific 6 (Gas6) and protein S (ProS1) are natural ligands for the homologous TAM (Tyro3, Axl, Mer) receptor tyrosine kinases (RTKs) [1]. Human ProS1 and Gas6 proteins share 43% amino acid sequence identity and are structurally similar. Both contain a series of γ-carboxyglutamic acid (Gla) residues at the *N*-terminal, a loop region, four EGF-like repeats, and a *C*-terminal sex hormone-binding globulin (SHBG)-like structure, which is composed of two globular laminin G-like (LG) domains with calcium-binding sites, LG1 and LG2 [2] (Fig. 1). The Gla residues are glutamic acid residues that have been post-translationally modified in a vitamin K-dependent manner. The clustering of Gla residues within this region, also referred to as the Gla domain, confers an enhanced binding affinity for phosphatidylserine-rich membrane surfaces, such as are present on apoptotic cells or activated platelets [3]. This feature is also necessary for ProS1 to negatively regulate coagulation by functioning as a critical cofactor for activated protein C and tissue factor pathway inhibitor (TFPI) [[4], [5], [6], [7]].

In humans, ProS1 circulates in the plasma at 350 nM concentration, of which 30%–40% exists in a free form whilst 60%–70% is in complex with the β-chain of C4b binding protein (C4BP), which precludes it from binding TAMs [8]. While Gas6 is expressed widely across many tissues and cell types [9], ProS1 is mainly synthesised by liver hepatocytes, although local tissue expression in different organs has also been detected. The TAM receptors possess in the extracellular (*N*-terminal) region a combination of two immunoglobulin (Ig)-like and two fibronectin type III domains, a single-pass transmembrane domain, and an intracellular region with intrinsic tyrosine kinase activity [10]. The Gas6/ProS1–TAM interaction is involved in a number of cellular biological processes including regulating the immune system and inflammation, cell survival, migration, proliferation and removal of apoptotic cells [[11], [12], [13]], and aberrant TAM signalling has also been implicated in cancers [14]. Gas6 activates all three TAM receptors but with different affinities: Axl > Tyro3>Mer [15], whereas ProS1 is a functional ligand for Tyro3 and Mer only [11]. The affinities of both TAM ligands for Tyro3 are greatly enhanced in the presence of phosphatidylserine in the membrane, with this effect being greater for Tyro3/Mer than for Axl [3,16]. The Gla domain in TAM ligands is able to coordinate calcium ions within a certain 3D fold, which mediates membrane interaction and enables both proteins to fully activate the TAM receptors [10,17].

The SHBG-like regions of both ProS1 and Gas6 contain a short segment encoded by exon IX of the *SHBG* gene, as well as two repeats that have sequence similarities with the globular domains of the α chain of laminin and laminin-related proteins, such as merosin and Drosophila crumbs [18,19]. The crystal structure of the Gas6 SHBG region reveals a V-shaped arrangement of LG domains stabilised by a calcium-binding site at their interface [2]. Approximately 60% of all residues under functional divergence are located in the SHBG-like region in both proteins. Within this region alone, computational modelling indicates notable differences in the electrostatic properties of the surfaces of the two proteins, revealing patches of different charge [20]. These could underlie specific differences between the two proteins in terms of their divergent intermolecular interactions. As regards TAM binding, both ProS1 and Gas6 SHBG regions interact directly with the Ig domains of the TAM receptors, causing receptor dimerisation and activation [1,18]. For Gas6, both LG1 and LG2 have been implicated in the interaction with the receptors [21,22]. The LG1 domain of Gas6 has been shown to directly bind Axl [23], whilst the LG2 domain contains a series of hydrophobic residues that may indirectly affect ligand-receptor binding [22]. In another study, the authors used an anti-Gas6 monoclonal antibody to show that the ligand-receptor binding region was localised at residues 403–414 within the Gas6 LG1 domain, in the region located close to the edge of the LG1 β-sandwich fold [23,24].

The Axl receptor contains two distinct Gas6-binding epitopes: a high-affinity site on its first Ig-like domain (Ig1) and a low-affinity site on the second Ig domain (Ig2). This may contribute to greater ligand affinity/specificity and explain why Gas6 alone can cause Axl homo-dimerisation in 2:2 stoichiometry and perhaps hetero-dimerisation amongst TAMs [15]. A crystal structure of a Gas6/Axl complex has also been presented and revealed that the LG1 domain of Gas6 makes two contacts with the Ig1 and Ig2 domains of Axl receptor [23]. A high-affinity interaction between Gas6 LG1 and Axl Ig1 domains is a major contact site. Receptor dimerisation then forms a 2:2 ligand/receptor complex assembly via a Gas6 LG1-Axl Ig2 minor contact [25]. In the major contact site, many charged residues were specified in both Axl and Gas6 that form part of interacting polar β-sheet surfaces. In comparison, a biophysical study determined that one site of the Gas6-Tyro3 interaction is localised close to the interface of the *N*-terminal Ig domains of the receptor, specifically a conserved surface patch on the Ig2 domain close to the inter-domain interface [26]. This is known as a minor contact site and is conserved across all three TAM receptors.

However, in contrast to the Gas6-Axl ligand-receptor pairing, relatively little is known about the structure-function relationships behind the role of ProS1 as a TAM ligand. One study using human-bovine ProS1 chimeras as an approach suggested several residues in the LG1 domain to be involved in the activation of Tyro3 [27], while others have suggested that the EGF-like domains might also be involved in the interaction [28]. Therefore, the precise region within the SHBG region that directly contacts the receptor remains to be determined. It is also of interest to investigate whether ProS1 has a similar distribution of charged residues to that in Gas6; this might explain why ProS1 is a preferential ligand for Mer and Tyro3 but not for Axl [2].

A structure comparison of Axl and Tyro3 reveals noticeable differences in their Ig1 domains, whereas their Ig2 domains are quite similar [26]. The Ig domains of human Tyro3 has been crystallised as a fragment in complex with Gas6 SHBG region [26] which together with binding studies, localised one site of the Gas6/Tyro3 interaction to the two Ig-like domains. However, further studies are required to investigate whether Tyro3 binds to TAM ligands in the same manner as Axl does with Gas6.

In this study, we have investigated the structure-function relationships surrounding the ProS1-Tyro3 ligand-receptor interaction. We have used chimeric and glycosylation mutant constructs to determine the specific TAM ligand properties inherent in those proteins versus the natural ligands [22,29]. We also investigated the downstream signalling pathways activated by the special ligand constructs in comparison to the native ligands. We have determined that the LG1 domain of ProS1 is necessary for its activation of Tyro3, whilst the equivalent domain in Gas6 is necessary for Axl activation.

## 2 Materials and methods

### 2.1 Cell culture

The SCC-25 human head and neck cancer cell line which expresses both Axl and Tyro3, and the MGH-U3 bladder cancer cell line, expressing only Tyro3 as TAM receptor [30], were both maintained in complete medium (DMEM + 10% FBS) at 37 °C in a humidified incubator with 5% CO_2_, as previously described [30].

### 2.2 Cell treatments

Cells were first serum-starved for 24 h, then treated for various time periods with TAM ligand proteins, which were recombinant human Gas6, developed in-house [31], and ProS1 (Cambridge Protein Works, Cambridge, UK) [30]. In addition, a set of recombinant ProS1/Gas6 chimeras were prepared [[32], [33], [34]]. These were all based on the ProS1 molecule, and included the N terminal portion of the protein up to the beginning of the *C*-terminal globular SHBG region, with each construct possessing a domain that was swapped with the corresponding domain from Gas6 as follows: entire Gas6 SHBG region (Val243-Ser635; chimera III), Gas6 LG1 domain only (Ser283-Val459; chimera I) or Gas6 LG2 domain only (Ser460-Ser635; chimera II) (Fig. 1a). We also generated five additional ProS1 variants (V) harbouring introduced *N*-linked glycosylation sites, using site-directed mutagenesis to make amino acid substitutions as follows: D253T (V1), L379T (V3), R404T (V5), G418 N (V6) and Q427 N/K429T (V7). All ProS1 variants were expressed to result in fully γ-carboxylated Gla domains as previously described [35]. SDS-PAGE analysis revealed a shift in molecular weight consistent with the presence of an additional *N*-linked glycosylation for variants V1, V3, V6 and V7. However, no shift was visible for V5, indicating that this variant does not bear a glycosylation but instead a single amino acid switch [36]. In ligand stimulation experiments, all protein S variants were added to cells at a final concentration of 7.5 nM for 9 min, as previously determined to be optimal for determination of TAM RTKs and intracellular signal pathway activation [27,30].

### 2.3 SDS–PAGE and western blotting

For cell lysis, ice-cold RIPA buffer (150 mM NaCl, 1% Triton X-100, 0.5% sodium deoxycholate, 0.1% SDS, 50 mM Tris pH 8.0) was used, supplemented with a cocktail of protease and phosphatase inhibitors. As previously described, SDS-PAGE and western blotting were performed on extracts using specific antibodies to detect activated, phosphorylated forms of Tyro3, Axl, Erk and Akt [30]. The primary antibodies recognising human proteins (and dilutions) used were: phospho-Axl (rabbit polyclonal; 1:500; R&D systems; AF2228), total Axl (goat polyclonal; 1:500; R&D systems; AF154), phospho-Erk (mouse monoclonal 1:1000; Cell Signaling Technology (CST); 9106), total Erk (rabbit monoclonal; 1:1000; CST; 9102), phospho-Akt (Ser473) (rabbit polyclonal 1:1000; CST; 9271), phospho-Tyro3 (rabbit polyclonal; 1:1000; Sigma; SAB4504621), total Tyro3 (rabbit monoclonal; 1:1000; CST; 5585), GAPDH (mouse monoclonal; 1:1000; Santa Cruz; sc-365062), β-actin (mouse monoclonal; 1:1000; CST; MAB8929). Membranes were first probed with phosphospecific antibodies, following which they were stripped and reprobed with the corresponding antibodies against the total proteins or GAPDH/actin as loading control proteins. Secondary antibodies used were anti-rabbit HRP (1:2000; Dako; P0339), anti-goat HRP (1:5000; Dako; P0449) and anti-mouse HRP (1:5000; Dako; P0447). The software *ImageJ* was used for densitometric quantification of Western blot band intensities [37].

### 2.4 Homology modelling and docking

Protein structures that had been experimentally determined through crystallisation were downloaded from the Protein Data Bank (PDB) [38]. Sequences were retrieved from the Uniprot Database [39]. Visualisation was carried out with PyMOL molecular graphics system (Schrödinger, LLC). Homology modelling was performed with the Swiss-Model web server [40] and protein-protein docking computations were carried out with pyDockWeb [41] and with HawkDock [42,43].

### 2.5 Modelled glycosylation sites

PDB files were manipulated with the Python scripts available in PDB-Tools [44] or the MayaChemTools Perl scripts [45]. Known and putative solvent exposed *N*-glycosylation sites were modelled in 3D using the GLYCAM-Web GAG Builder service and the high mannose oligosaccharide library [46]. Electrostatic calculations were computed with the Adaptive Poisson-Boltzmann Solver (APBS) [47] and the Protein Continuum Electrostatics server [48]. The two laminin LG domains of human ProS1 were built using Swiss-Model and the crystal structure of the equivalent domains of human Gas6 in complex with the two *N*-terminal Ig domains of Axl (PDB file: 2c5d) [23]. The sequence identity between human ProS1 and human Gas6 is around 43% and there are no major insertions or deletions between the two proteins. Three known *N*-glycosylated sites are present in the LG2 domain of ProS1 (N458, N468, N489); these were grafted using the GLYCAM-Web GAG Builder service. Further, as two short loops were missing in the X-ray structure of Gas6, they were built using the Swiss-Model server. Gas6 N420 is glycosylated and two sugar rings have been defined in the experimental structure. The X-ray structure of Tyro3 containing two Ig domains (Ig-like C2-type 1 and Ig-like C2-type 2) (PDB file: 1rhf) was used for the docking computations. For Tyro3, putative *N*-glycosylation sites were modelled in 3D using the GLYCAM-Web GAG Builder service. Similarly, putative *N*-glycosylation sites were modelled on both Axl Ig domains using the X-ray structure of Axl extracted from the PDB file 2c5d.

### 2.6 Statistical analysis

All experimental data from cells is expressed as mean ± SEM, obtained from a minimum of 3 independent experiments, with each treatment constituting multiple replicate wells per condition. Quantitative data were subjected to analysis by analysis of variance (ANOVA) and *post-hoc* Tukey test for multiple comparisons with one control group. Statistical analysis and preparations of graphs were performed using Prism software (GraphPad Software Inc, San Diego, CA). The degree of statistical significance is indicated by symbols in the figures and accompanying legends, and *p* < 0.05 was considered as statistically significant.

## 3 Results

### 3.1 The LG1 domains of ProS1 and Gas6 are necessary and sufficient for activation of, respectively, Tyro3-Erk and Axl-Akt, signalling in SCC-25 cells

To begin with, SCC-25 cells were used as they were responsive to both ProS1 and Gas6 ligands via Tyro3 and Axl receptors respectively, and furthermore do not express ProS1, as we have previously shown [30]. Out of the chimeras used (Fig. 1a and Fig. S1), only chimera II (Ch II) (ProS1 LG1; Gas6 LG2) significantly stimulated Tyro3 phosphorylation to the same extent as wildtype ProS1 (Fig. 1b). Moreover, this effect on pTyro3 by both ligands was mirrored in their concurrent stimulation of Erk kinase phosphorylation (Fig. 1b). However, no such effect on Tyro3-Erk was observed by Gas6 and the other chimeras, although Gas6 did weakly stimulate Tyro3.

In contrast, the chimeric proteins that at a minimum contained the Gas6 LG1 domain were able to stimulate Axl phosphorylation to the same extent as wildtype Gas6 (Fig. 1c). These were chimera I (Ch I) (Gas6 LG1; ProS1 LG2) and chimera III (Ch III) (whole Gas6 SHBG region). This effect on Axl was mirrored in their activation of Akt kinase phosphorylation, whereas no significant stimulation occurred with ProS1 or chimera II (Fig. 1c).

Therefore, for both ProS1 and Gas6, the LG1 domain is necessary as well as minimally sufficient to enable the protein to activate its respective TAM receptor and associated downstream signalling pathways.

### 3.2 In cells expressing Tyro3 as sole TAM receptor, chimeras containing the LG1 domains of ProS1 or Gas6 act as the respective natural ligands

**Fig. 2:**
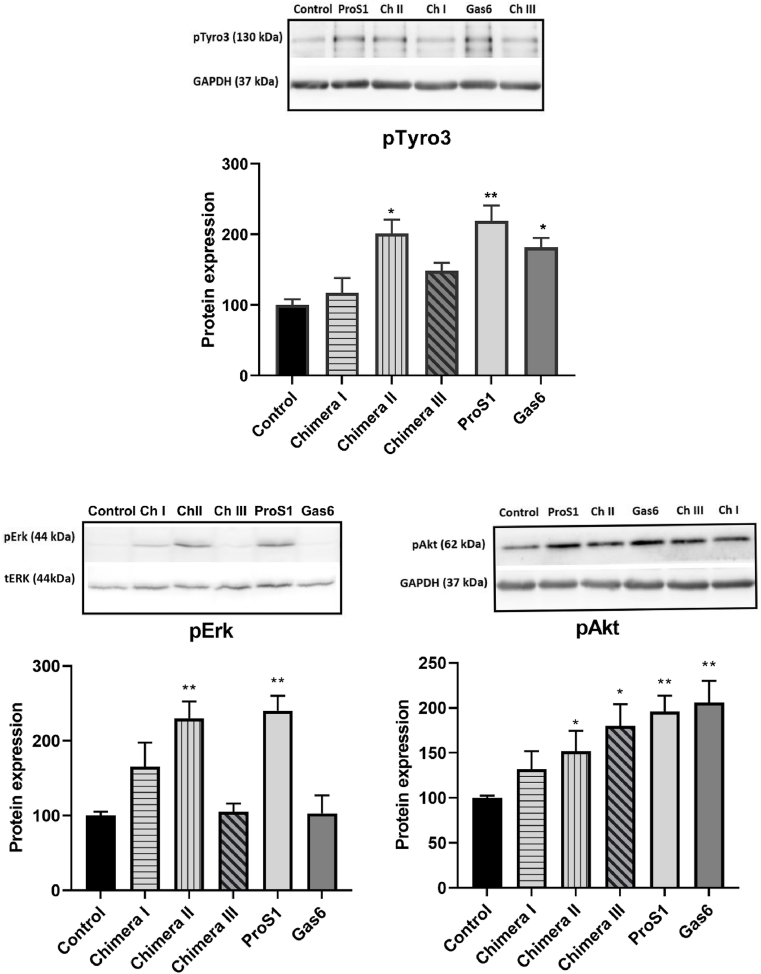
The effects of ProS1, Gas6 and chimeras stimulation on Tyro3 receptor and coupled downstream signalling molecule activation in MGH-U3 cells (expressing Tyro3 only). Western blots show levels of phosphorylated of Tyro3 (pTyro3) and downstream signalling kinases, pErk and pAkt, after stimulation by ProS1, Gas6 and chimeras (7.5 nM) for 9 min. Accompanying graphs show protein quantification by densitometric analysis of bands (n = 4 separate experiments). Data as mean ± SEM expression for each phosphoprotein was normalized against the total protein/loading control (tERK, GAPDH). ANOVA with Tukey's multiple comparison *post-hoc* analysis; **p < 0.01, *p < 0.05 versus control (untreated). Although the sample loading order varies across blots, the quantification bar charts are presented in the same order for consistency.

We have previously shown that in MGH-U3 cells, which express Tyro3 as sole TAM receptor, Tyro3 has a broader receptivity and is also activated by Gas6 in addition to ProS1 [10]. However, in these cells, Gas6 stimulates Akt but not Erk, whereas ProS1 activates both Erk and Akt. Here, chimera II (ProS1 LG1; Gas6 LG2) significantly activated Tyro3 to the same extent as ProS1, whilst a weak but variable stimulation was observed with chimera III (whole Gas6 SHBG region) and Gas6 (Fig. 2). In addition, chimera II was the sole stimulator, alongside ProS1, of Erk phosphorylation. Furthermore, all recombinant proteins caused increased Akt phosphorylation in MGH-U3 cells. Therefore, Tyro3 sole expression affords the same versatility in sensitivity to the chimeric TAM ligands as exists towards the wildtype ligands, according to which ligand the chimera most resembles through its minimal LG1 domain.

### 3.3 Identification of a major TAM receptor contact site in the LG1 domain of Gas6

**Fig. 3:**
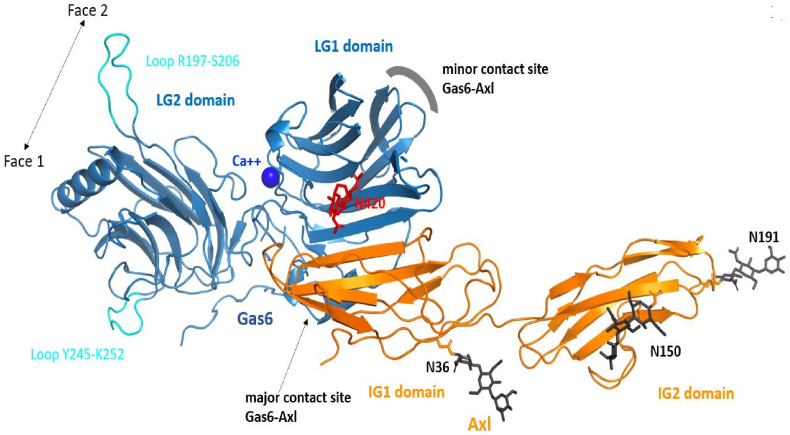
Major contact site between Gas6 and Axl. The experimental structure of the Gas6-Axl complex is shown (PDB ID: 2c5d). The proteins are in a cartoon representation, the LG domains of Gas6 are in blue while the Ig domains of Axl are in orange. Two missing loops in Gas6 were built (cyan). The Gas6 calcium ion is shown as a sphere while two sugar rings are noticed in the X-ray structure (red). Three putative *N*-glycosylation sites are found in this region of Axl, the glycans were grafted onto the X-ray structure and only three sugar rings were kept. The minor contact site between Axl and Gas6 is highlighted with a thick grey line but the protein interaction is not shown for greater clarity as the image in this orientation pertains to the major contact. (For interpretation of the references to colour in this figure legend, the reader is referred to the Web version of this article.)

Following the experimental evidence that the LG1 domain of TAM ligands is necessary for TAM functional activation, we set out to propose a model of the Tyro3-ProS1 complex. We first investigated the experimental structure of Gas6 co-crystallised with Axl. The crystal structure of Gas6 in complex with Axl has been reported by Sasaki et al. [23]. In this protein-protein complex, two interaction sites were found. The major contact site involves the LG1 domain of Gas6 and essentially the first immunoglobulin (Ig1) domain of Axl (Fig. 3). A minor contact site was also noted, which involved another region of the Gas6 LG1 domain and both Ig domains of Axl.

In Gas6, a large electropositive groove is present on what we refer to “Face 1” of the LG1 domain, whilst a clear electronegative region is present on the Ig1 domain of Axl, exactly within the region of the major contact site (Fig. S2). Thus, in this region, the two proteins are likely driven together through electrostatic interactions, further stabilised by polar contacts and hydrophobic interactions, which become buried upon formation of the final complex. Face 2 of Gas6 is much more electronegative, especially on the LG2 domain, while two small electropositive regions are seen on the LG1 domain (Fig. S2).

Using two online services, pyDockWeb and HawkDock, we then docked Axl domains Ig1 and Ig2 onto the crystal structure of Gas6 (to which we added two missing loops that are far away from the protein-protein interface using the Swiss-Model web server) (Fig. S3). Sugar molecules were removed and the full surface of Gas6 was explored. Both docking engines predicted very accurately the major Gas6-Axl contact site that was experimentally determined. Axl was also positioned in the region of the minor contact site on Gas6 but such complexes were ranked around position 100, consistent with the much smaller contact areas observed in the crystal structure.

### 3.4 Modelling of the interaction between ProS1 LG1 and Tyro3 Ig1 domains

**Fig. 4:**
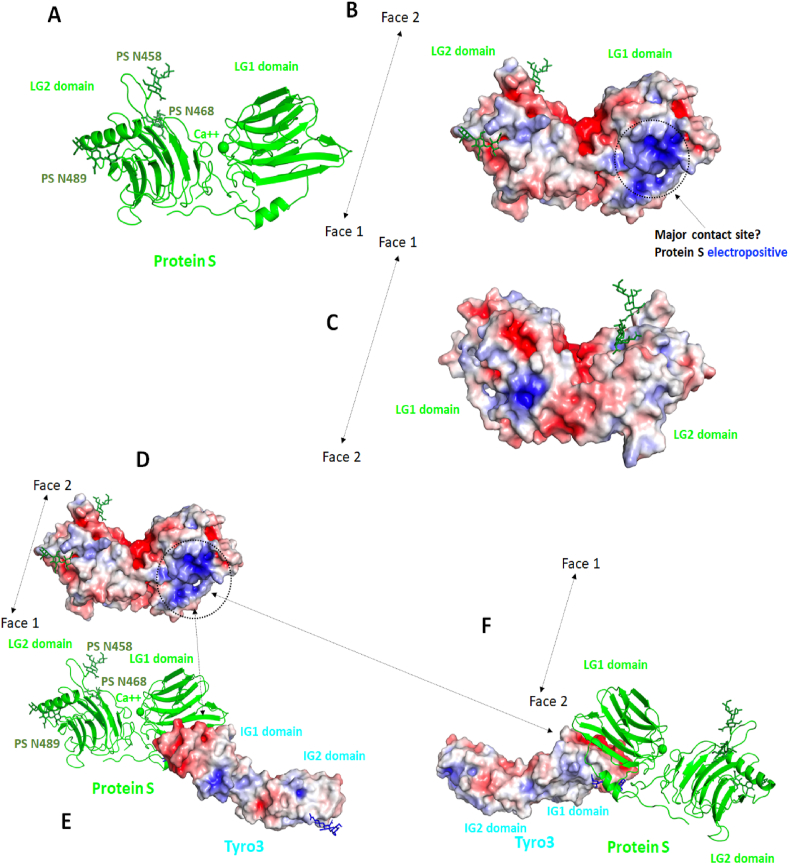
Negative and positive electrostatic potentials mapped onto the molecular surface of predicted ProS1 structure and Tyro3. (a) The predicted structure for this region of ProS1 is shown in a cartoon representation. The calcium ion is shown as a green sphere and the three *N*-glycosylation sites are highlighted. (b) Negative and positive electrostatic potentials mapped onto the molecular surface of the ProS1 model. Face 1 is visible in this orientation. The potentials are on a −5, +5 red-white-blue colour map in units of kJ/mol/e. A major electropositive region is observed in the LG1 domain of ProS1. (c) The other side of ProS1 is shown (Face 2). (d) ProS1 electrostatic potentials; Face 1. The negative regions are in red and the positive areas in blue. The potentials are on a −5, +5 red-white-blue colour map in units of kJ/mol/e. (e) Tyro3 electrostatic potentials. The orientation is similar to the one shown in A. A key electronegative surface is noticed in the Ig1 domain of Tyro3, next to the electropositive surface of ProS1. (f) View of the other face (Face 2). (For interpretation of the references to colour in this figure legend, the reader is referred to the Web version of this article.)

Modelling ProS1 using the experimental structure of Gas6 was facilitated by the high sequence identity between this region of the two proteins. The new ProS1 model displays some differences in the orientation of the two LG domains and for some loop regions as compared to a previous model that we developed some years ago using the individual LG domains of laminin and plasma SHBG aligned onto the structure of a laminin structure that contained two LG domains [49]. The RMSD for the backbone atoms between the two models is about 4 Å. We then investigated the modelled ProS1 structure, the experimental structures of Gas6, Axl and Tyro3 and docked Tyro3 onto the ProS1 model (Fig. 4).

In the new ProS1 model, the three known glycosylation sites are solvent exposed, with N458 and N468 located on Face 2 of the LG2 domain and N489 presents on Face 1 of the LG2 domain (Fig. 4). The calcium-binding site is essentially located in the LG1 domain near the LG1/LG2 interface. Electrostatic computations were carried out for this region of ProS1. A clear electropositive region is observed on Face 1 of the ProS1 LG1 domain, in the same area as the electropositive region in Gas6 and in the exact area of the major contact site between Gas6 and Axl (Fig. 4). Face 2 of ProS1 tends to be neutral or negatively charged with only a small electropositive region located in the LG1 domain.

**Fig. 5:**
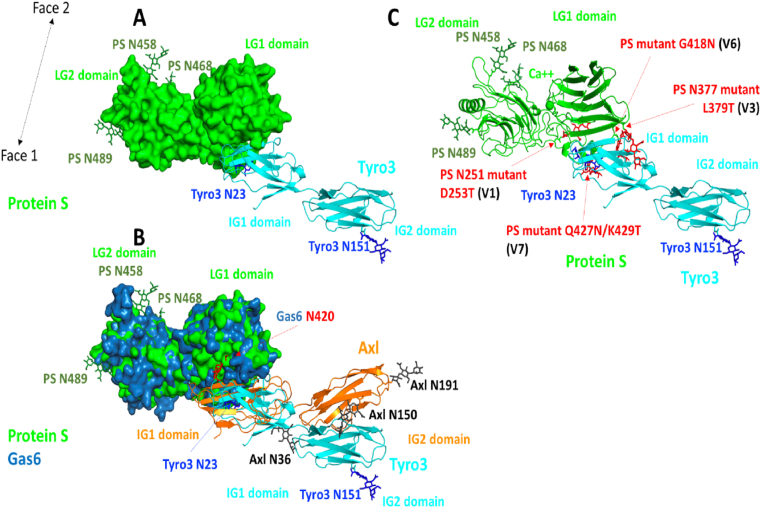
ProS1-Tyro3 docking and structural analysis. (a) The best predicted binding pose for Tyro3 (cyan) on ProS1 (green) is shown. The putative *N*-glycosylation sites in Tyro3 are shown in dark blue whilst the glycosylated sites in ProS1 are in dark green. (b) The docked ProS1-Tyro3 model is superimposed onto the X-ray structure of the Gas6-Axl complex. (c) Several additional glycosylation sites were engineered on the surface of ProS1, as present on a set of mutant ProS1 (PS) constructs generated for cell stimulation experiments. Three sugar rings were added at each site. One of the variants, V5, is likely not to bear a glycosylation but instead bears a single amino acid switch; hence it is not highlighted in the figure. None of these sites seems to interfere with Tyro3 in the proposed docked orientation. (For interpretation of the references to colour in this figure legend, the reader is referred to the Web version of this article.)

We then docked the experimental Tyro3 Ig1-Ig2 domain structure [26] onto the surface of the LG domains of ProS1. Here again, all sugar molecules were deleted for the docking but used afterwards in an attempt to discriminate the poses, as glycosylated Asn residues cannot be present right in the middle of a protein-protein interface. Further, it has been observed that the two Ig domains of Axl interacts with Gas6 in a fully extended fashion whereas the Tyro3 crystal structure is slightly bent [26]. The two docking engines predicted Tyro3 to fit onto the electropositive region presents on Face 1 of the LG1 domain of ProS1 (Fig. 4). To investigate further this particular orientation of Tyro3 on the surface of ProS1, we investigated the electrostatic potentials mapped onto the molecular surfaces of both proteins (Fig. 4). When looking at Face 1 of ProS1, it is clear that the LG1 domain is mainly electropositive in a region that is equivalent to the main contact site present on Gas6. Similarly, although the Tyro3 structure has some differences with that of Axl, the Ig1 domain of Tyro3 predicted to be in contact with ProS1 is nevertheless electronegative and is indeed the main electronegative region in this area of Tyro3. The top HawkDock predicted binding energy for Tyro3 on the surface of ProS1 is also seen in (Fig. 5). pyDockWeb also found this very same orientation, but it was not ranked at the first position.

### 3.5 Addition of novel glycosylations to the LG1 domain of ProS1 does not perturb its activation of Tyro3 and associated downstream signalling

**Fig. 6:**
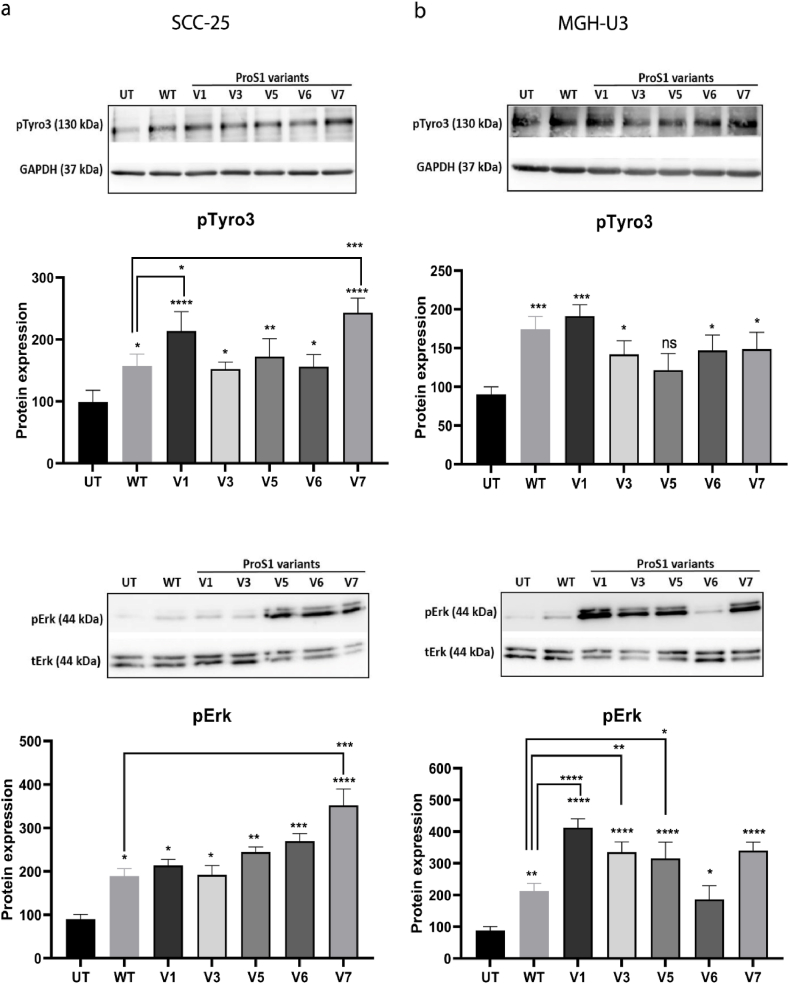
The **e**ffects of ProS1 glycosylation mutant protein variants on stimulation of Tyro3 receptor and Erk kinase in SCC-25 (a) and MGH-U3 (b) cells. Western blots showing levels of phosphorylated Tyro3 (pTyro3) and Erk (pErk) proteins after stimulation by wildtype ProS1 and the variants (7.5 nM for 9 min). The following mutant variants of ProS1 were used: D253T (V1), L379T (V3), R404T (V5), G418 N (V6) and Q427 N/K429T (V7). Accompanying graphs show protein quantification by densitometric analysis of bands (n = 4 separate experiments). Data as mean ± SEM expression for each phosphoprotein was normalized against the total protein/loading control; ANOVA with Tukey's multiple comparison *post-hoc* analysis; ****p < 0.0001, ***p < 0.001, **p < 0.01, *p < 0.05, versus control (untreated) or for comparisons indicated by lines.

To further screen for a functional interaction site for Tyro3 on the LG1 domain of ProS1, five recombinant variants of ProS1 protein were generated. In four of these, novel *N*-linked glycosylation sites were inserted within the LG1 domain through amino acid substitutions (Fig. 5c). Along with wildtype ProS1, these variants were added to both SCC-25 and MGH-U3 cancer cell lines which served as readouts for Tyro3 and Tyro3-dependent Erk kinase activation. All of the ProS1 glycosylation mutant proteins significantly stimulated Tyro3 and Erk phosphorylation in both cell lines, to at least the same extent as wildtype ProS1 (Fig. 6). Furthermore, two of the mutant proteins (V1 and V7) stimulated Tyro3 and Erk to a significantly greater extent than wildtype ProS1. This could be due to a ligand conformation that binds the receptor more strongly and/or a different kinetics of Tyro3 activation, where greater activation is achieved with these two variants at the particular time period analysed. Therefore, these results show that none of the inserted glycosylation sites overlapped with the main contact site predicted by the docking.

## 4 Discussion

The homologous vitamin K-dependent proteins ProS1 and Gas6 are natural ligands for the TAM subfamily of RTKs, although with different receptor affinities. Gas6 activates all three TAM receptors, with greatest affinity for Axl followed by Tyro3 then Mer, whereas ProS1 is a ligand for Tyro3 and Mer only [11]. We have shown that ProS1 is a preferred ligand for Tyro3 over Gas6 in human cancer cells expressing both Tyro3 and Axl receptors [30], as well as identified Tyro3 signaling pathways that progress the cell cycle and support survival in cancer cells [50]. However, while there is insight into the structure-function relationships that exist for the Gas6-Axl interaction [23], no such knowledge exists on the nature of the ProS1-Tyro3 interaction. In the present study, we have used a combination of mutagenesis, domain swapping, cell biology and structural biology to determine that the LG1 domain within the C terminal SHBG-like region of ProS1 is essential for its physical interaction with, and activation of, the Tyro3 receptor.

The domain organisations of ProS1 and Gas6 are homologous and the *C*-terminal SHBG region, consisting of two LG domains, has previously been shown to play a crucial role in the stimulation of the TAM receptor. In order to localise the protein-protein interaction interface within the SHBG region of ProS1 as a Tyro3 ligand, we used a set of human ProS1 chimeric constructs in which one or both of the LG domains of the SHBG region of ProS1 was replaced by the corresponding domain of Gas6. These were then used to compare with the wildtype proteins for their potential stimulatory effects in two human cancer cell lines that contained different TAM receptor combinations [30]. In SCC-25 cells, which express both Tyro3 and Axl, only the chimeric protein that contained the ProS1 LG1 domain was, alongside wildtype ProS1, uniquely able to activate Tyro3 receptor. Conversely, chimeras that contained minimally the Gas6 LG1 domain were, alongside wildtype Gas6, uniquely able to activate Axl receptor. These results therefore showed that the LG1 domain within each ligand is essential for its activation of its respective TAM receptor.

We also used MGH-U3 cells, expressing Tyro3 as sole TAM receptor, in which Gas6 is able, as a weaker ligand, to co-opt Tyro3 to activate Akt signalling [30]. In these cells, the mutual exclusivity of ProS1 vs Gas6 was further evidenced by the observation that the chimera containing Gas6 LG1 domain was uniquely unable to stimulate Tyro3, whereas the chimera containing the entire Gas6 SHBG region was, alongside wildtype Gas6, able to do so. This additional observation in these cells indicate that the relatively weak stimulation of Tyro3 by Gas6 is only possible either through its entire SHBG region or otherwise that the LG2 domain is minimally necessary, but not sufficient, for Tyro3 activation. Therefore, these data indicate that the LG1 domain contains critical residues for the interaction with Tyro3.

The necessity of the ProS1 LG1 domain for TAM receptor stimulation observed here is matched by other studies showing a similar importance of this part of ProS1 for protein interactions that regulate roles in other physiological contexts, including the blood coagulation and complement cascades. For example, the chimeras used in the present study have previously been characterised for their interactions with the complement regulatory protein C4BP [51]. It was determined that both LG domains of ProS1 were involved in the interaction with C4BP and that full affinity binding was dependent on contributions from both domains, although the LG1 domain was a more efficient interactor than LG2. In contrast, the Gas6 SHBG region was a weak interactor with C4BP. This again highlights the distinct differences between the diverse set of protein-protein interactions involving ProS1 whereas Gas6 appears to have a more restricted interactome. Furthermore, we have also used the ProS1/Gas6 chimeras to functionally characterise ProS1 interaction with TFPI, for which ProS1 is a cofactor, accelerating the inhibition of activated factor X (FXa) [32]. We observed that the chimera containing ProS1 LG1 was equally effective as wildtype ProS1 at enhancing TFPI activity whilst, in contrast, chimeras with Gas6 LG1 or the entire Gas6 SHBG region had minimal effects.

Therefore, the above studies have highlighted the ProS1 LG1 domain as a prominent interface for protein-protein interactions in diverse biological settings ranging from blood coagulation, complement regulation and, as shown here, RTK activation. However, this does not preclude other domains from taking precedence in other interactions. For example, the ProS1/Gas6 chimeras were used to investigate the structure-function relationships behind the role of ProS1 as cofactor for the activated protein C (APC)-mediated proteolytic cleavage of coagulantion factors Va and factor VIIIa [34]. In a coagulation assay, the chimera containing Gas6 LG1 (with ProS1 LG2) was as efficient as wildtype ProS1, whereas the chimera containing ProS1 LG1/Gas6 LG2 was less effective. Furthermore, ProS1 LG2 was indispensable for the synergistic cofactor activity of factor V in the inactivation of factor VIIIa, as well as also being most important for the degradation of FVa. Therefore, the two LG domains of ProS1 appear to play distinct role in different contexts regarding protein interaction complexes.

In keeping with the domain dependence of the ProS1-Tyro3 interaction, our results also showed that the chimeric proteins containing at a minimum the Gas6 LG1 domain were able to stimulate Axl, and downstream Akt, phosphorylation with equal strength to wildtype Gas6. These findings are in accordance with the known experimental structural model of the Gas6-Axl interaction, which occurs through at minimum a major contact between Gas6 LG1 and the Ig1 domain of Axl [23].

Our model of the ProS1-Tyro3 interaction was based on the structure of the Gas6-Axl interaction interface and compared with the experimentally determined crystal structure [23]. Gas6 is glycosylated at N420 and two sugar rings are visible in the experimental structure; however, this part of the glycan does not interact with Axl. On the Axl side, three Asn residues could be glycosylated but these sites are distant from the protein-protein binding regions and are not known to play a role in the interaction [24]. A complementarity of charged residues was observed between the two proteins in the major Gas6-Axl binding site, which would be further stabilised by polar contacts and interactions between hydrophobic residues. In fact, the best binding energy and thus the first pose for Gas6-Axl generated by HawkDock superimposed almost perfectly onto the experimental structure (Fig. S3). pyDockWeb also predicted a pose very close to the X-ray structure but ranked this complex at the second best predicted energy value. However, the lowest energy orientation of Axl in pyDockWeb was not compatible with the glycosylation within Gas6 at N420 and we thus rejected it.

Our new model of ProS1 enabled display of some important solvent exposed regions, such as possible binding sites for a complement protein, location of the calcium binding site, and of the glycosylated regions. Moreover, as the sequence identity between ProS1 and plasma SHBG or laminin is much lower than for Gas6, the new model should therefore be more accurate for performing molecular docking experiments. In addition, despite some structural differences, the Ig domains of Axl and Tyro3 are similarly formed by beta-strands connected by loops, and the RMSD between their Ig1 domains is around 2.5 Å. In Tyro3, there are two putative *N*-glycosylation sites, one at N23 in the first Ig domain and one at N151 in the second Ig domain. It is also interesting to note that, despite some structural differences and the fact that the Tyro3 Ig structure is slightly bent as compared to Axl, the best ranked position for Tyro3 was highly similar to the position of the Axl Ig1 main contact site with Gas6. In fact, the bend in between the two domains of Tyro3 did not interfere with the docking computations as the main contact areas were located on the Tyro3 Ig1 domain and on the LG1 domain of ProS1. In this orientation, the putative Tyro3 glycosylation sites at N23 and N151 do not impede the interaction with ProS1. N23 is close to the interface, somewhat like Gas6 N420, but the complex could be formed *in silico* without problems as it is not directly at the interface. In addition, we tested five recombinant mutant variants of ProS1 protein, each of which harboured novel putative glycosylation sites in the LG1 domain (although four of these were deemed to actually possess a novel glycosylation). All of these ProS1 variants stimulated Tyro3 and Erk phosphorylation in cells to at least the same extent as wildtype ProS1, hence none of them perturbed the ProS1-Tyro3 interaction. Some of the extra glycosylation sites are close to the interface, but none of them were within the main contact site predicted by the docking. Therefore, these mutants exclude the regions in which they appear as being direct interfaces for the ProS1-Tyro3 interaction, hence further supporting the modelled contact region.

We therefore hypothesise that, as in the case of the main contact site of Gas6-Axl, the ProS1 LG1-Tyro3 Ig1 interaction is driven by long range electrostatic interactions, and as the two proteins get close in space, can be further stabilised by hydrophobic and polar contacts, which then become buried in this predicted orientation. As such, the suggested overall similarity in terms of binding between Gas6/Axl and Gas6/Tyro3 (not shown) [23] in the main contact site is fully supported by our docking computations, structural analysis of the interfaces in terms of electrostatic and hydrophobic complementarity and in line with the results of our mutagenesis data. However, we do not know at this stage if Tyro3 could also have contact with a region in ProS1 equivalent to the minor contact site in Gas6. As the minor contact site is conserved across all three TAM receptors, we can speculate that the ProS1-Tyro3 interaction could also involve the formation of a circular 2:2 complex featuring continuous beta-sheets across both types of contacts. Future modelling-led studies using e.g. mutagenesis would be required to probe key regions of interest within the ProS1 LG1 domain and both Tyro3 Ig1 and Ig2 domains to determine this.

## 5 Conclusions

In summary, the results of this study have elucidated for the first time the location and physical nature of the interacting regions within ProS1 as ligand and Tyro3 as receptor. Furthermore, given the similarity between the Gas6-Axl and ProS1-Tyro3 contact sites, it is therefore likely that a similar nature of contact exists for Mer when interacting with both ligands, although this remains to be studied. In addition, this study further expands the knowledge on the diverse repertoire of protein-protein interactions involving ProS1, reflecting its diverse roles in regulation of blood coagulation, complement activation and the various cellular functions mediated by TAM RTKs.

## Credit author statement

Conceptualisation, SH; Methodology, NK, JA, AT, LC, NS, BV; Data analysis and validation, SH, NK, JA, AT, LC, NS, BV; Resources, SH, JA, BV; Data Curation, NK, BV; Writing—original draft preparation, NK, SH, BV; Writing—review & editing, SH, NK, JA, AT, LC, NS, BV; Funding Acquisition, SH. All authors read and approved the final manuscript.

## Funding

This research received no external funding.

## Declaration of competing interest

The authors declare no conflict of interest.

## Data Availability

Data will be made available on request.
