# Supplementary material for: The first laminin G-like domain of protein S is essential for binding and activation of Tyro3 receptor and intracellular signalling
Source: Biochem Biophys Rep. 2022 Apr 28;30:101263. doi: 10.1016/j.bbrep.2022.101263 (PMC9065593; doi:10.1016/j.bbrep.2022.101263)
Supplement: Multimedia component 1 [file mmc1.pdf]

Al Kafri *et al*. **The first laminin G-like domain of protein S is essential for binding and activation of Tyro3 receptor and intracellular signalling.**

Western blot images

Uncropped chemiluminescence images of western blot membranes are shown below. Membranes from some gels were either stripped and reprobed for the total versions of the phosphoproteins, or else probed in separate parts to detect different proteins of interest in their respective mol wt ranges. After incubation with the relevant antibodies, membranes were exposed for development for the appropriate lengths of time for each protein. Sections of membranes within certain mol wt ranges that are not displayed here were used for probing for other proteins not relevant to this investigation. In some membranes, mol wt markers are not always visible at the exposures used; therefore, the images are reproduced with darker exposures, and arrows are used to show the sizes of visible bands.

Figure 1b

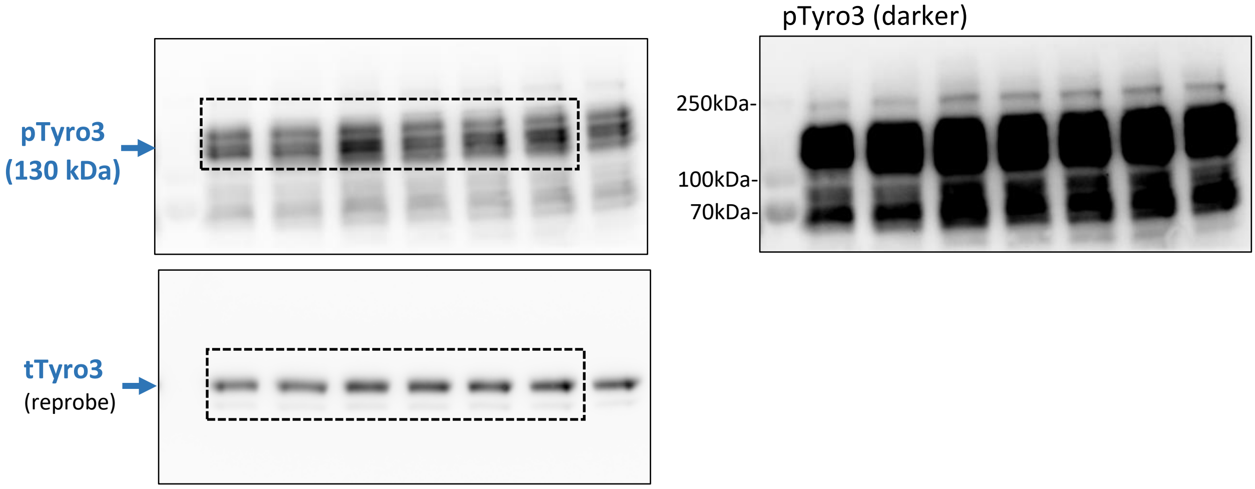

Membrane showing phospho-Tyro3 (pTyro3), then stripped and reprobed for total Tyro3 (tTyro3). Box with dotted line edges outlines the part of the blot displayed in the figure of the paper that includes the samples relevant to the experiment. A darker exposure of the pTyro3 blot is also shown to reveal the molecular weight markers.

Figure 1b (lower)

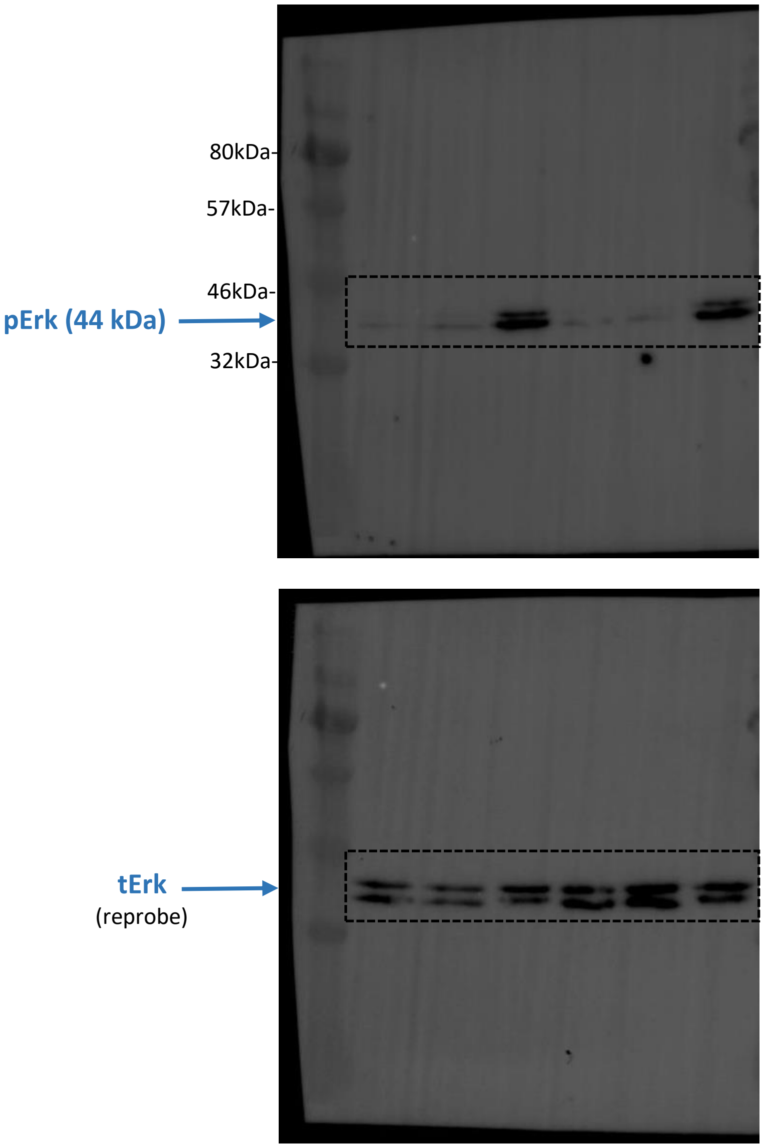

Membrane showing phospho-Erk (pErk) then stripped and reprobed for total Erk (tErk). Box with dotted line edges outlines the part of the blot displayed in the figure of the paper that includes the samples relevant to the experiment.

Figure 1c

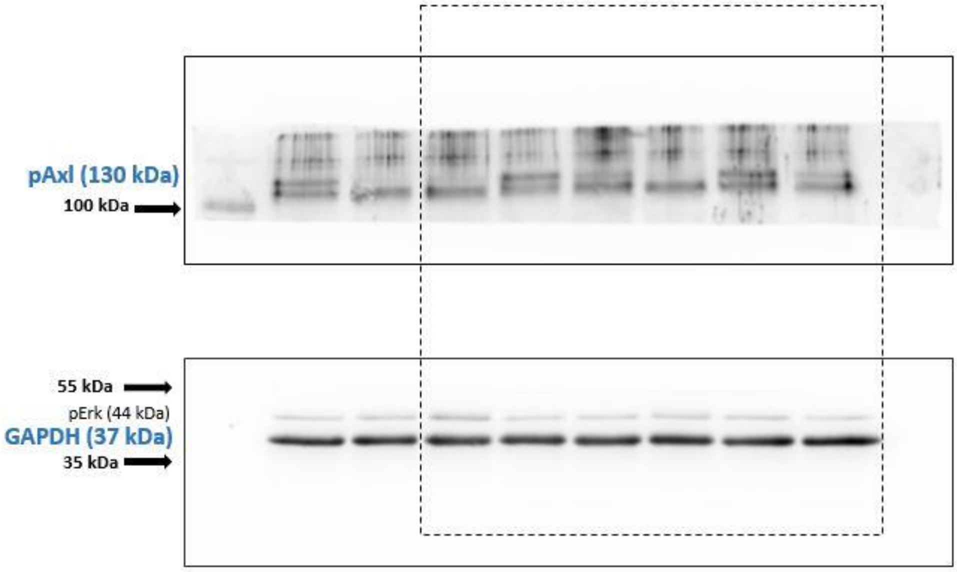

Membranes from same gel showing pAxl and GAPDH. Box with dotted line edges outlines the part of the blot displayed in the figure of the paper that includes the samples relevant to the experiment.

Figure 1c (lower)

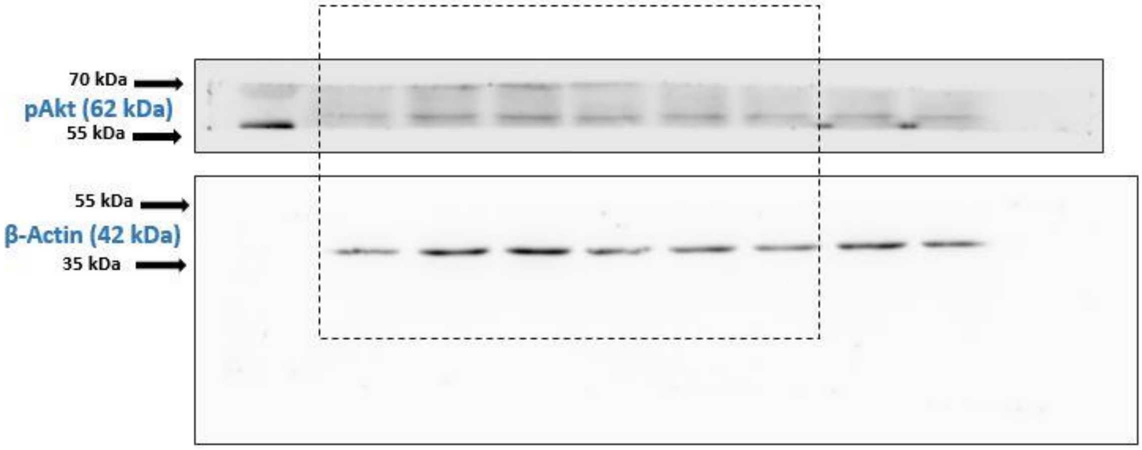

Membranes from same gel showing pAkt and β-actin. Box with dotted line edges outlines the part of the blot displayed in the figure of the paper that includes the samples relevant to the experiment.

Figure 2

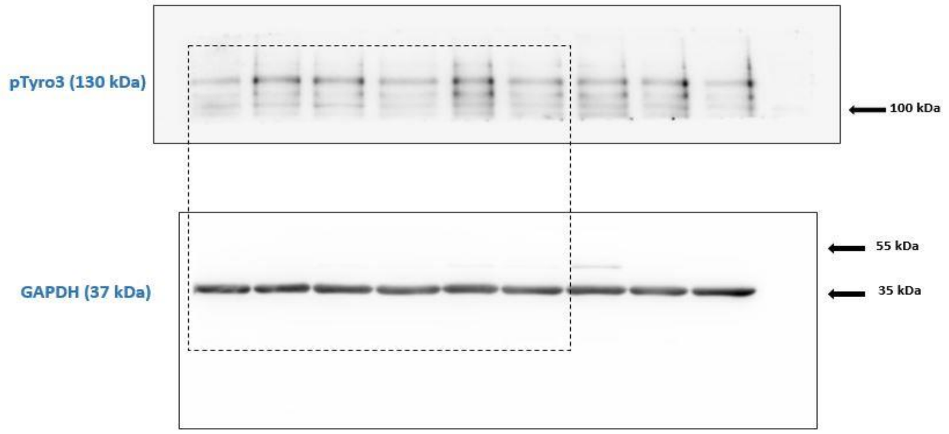

Membranes from same gel showing pTyro3 and GAPDH. Box with dotted line edges outlines the part of the blot displayed in the figure of the paper that includes the samples relevant to the experiment.

Figure 2 (pErk)

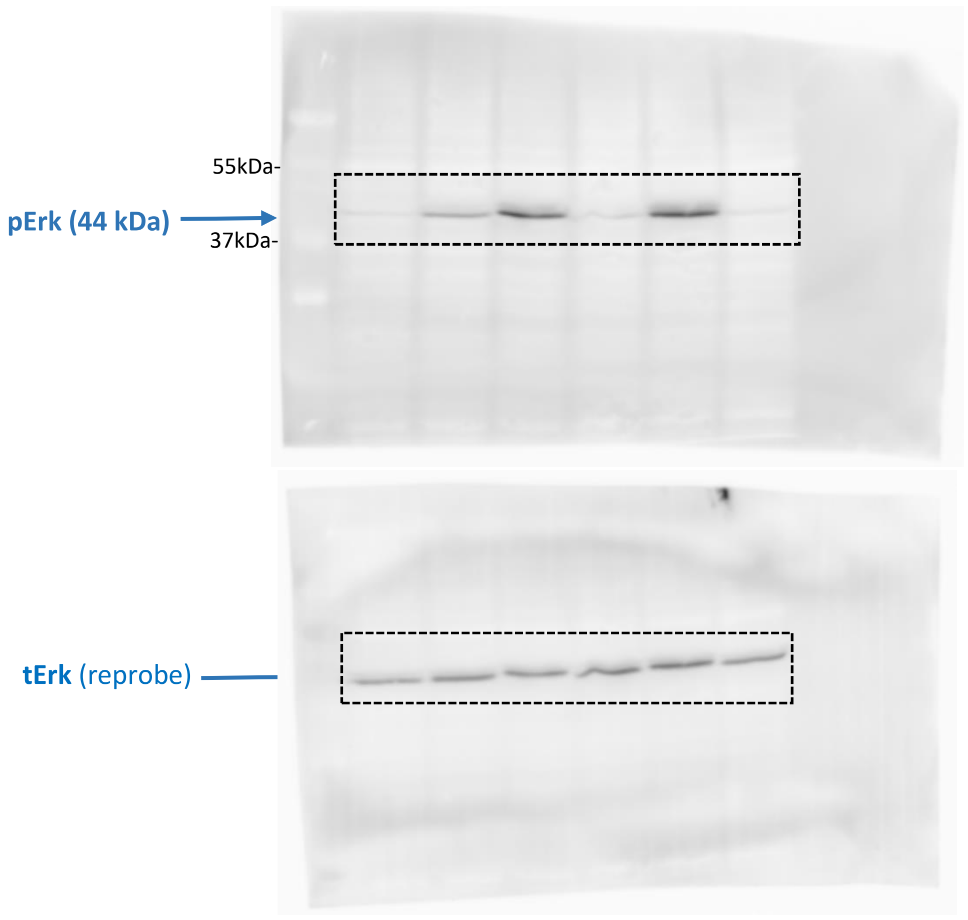

Membrane showing phospho-Erk (pErk) then stripped and reprobed for total Erk (tErk). Box with dotted line edges outlines the part of the image displayed in the Figure in the main manuscript.

Figure 2 (pAkt)

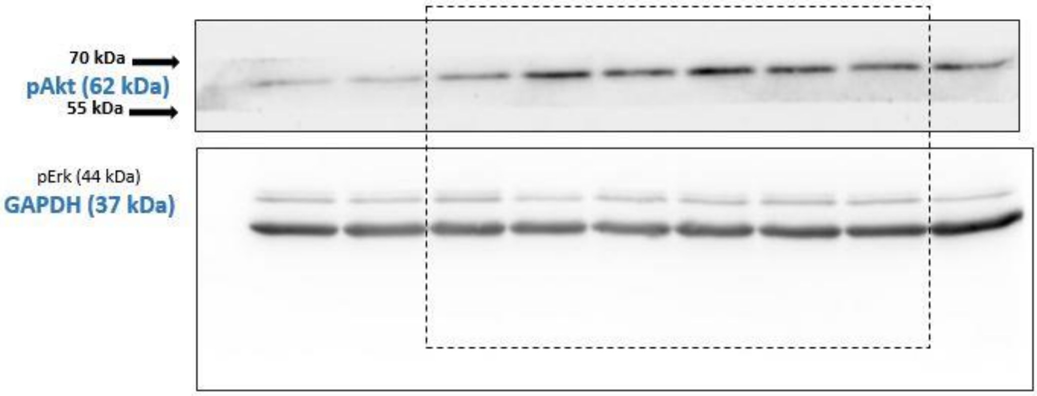

Membranes from same gel showing pAkt and GAPDH. Box with dotted line edges outlines the part of the blot displayed in the figure of the paper that includes the samples relevant to the experiment. Also wealy visible on the lower membrane are bands for pErk first probed for prior to reprobing for GAPDH without stripping.

Figure 6a

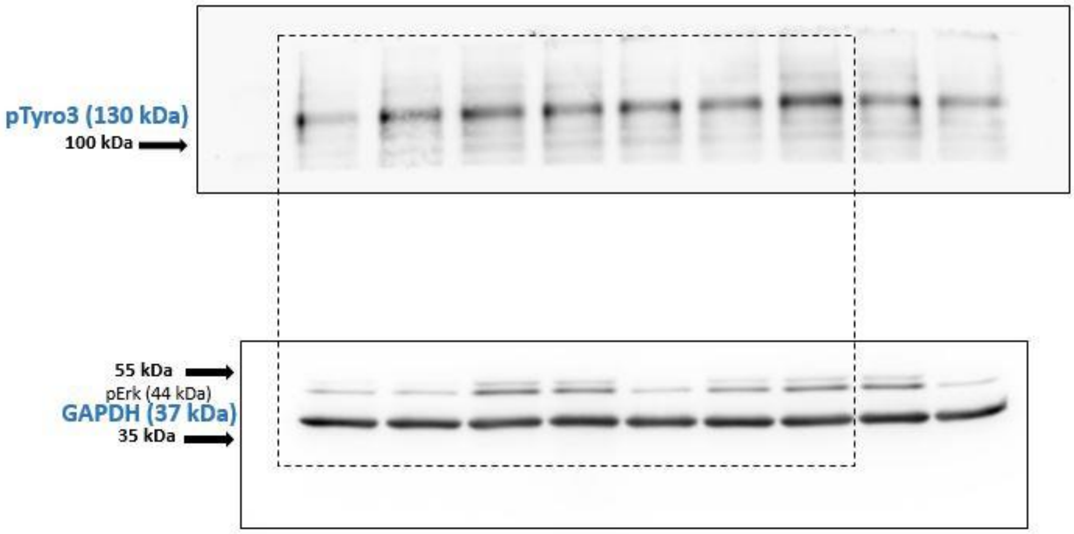

Membranes from same gel showing pTyro3 and GAPDH. Box with dotted line edges outlines the part of the blot displayed in the figure of the paper that includes the samples relevant to the experiment.

Figure 6a

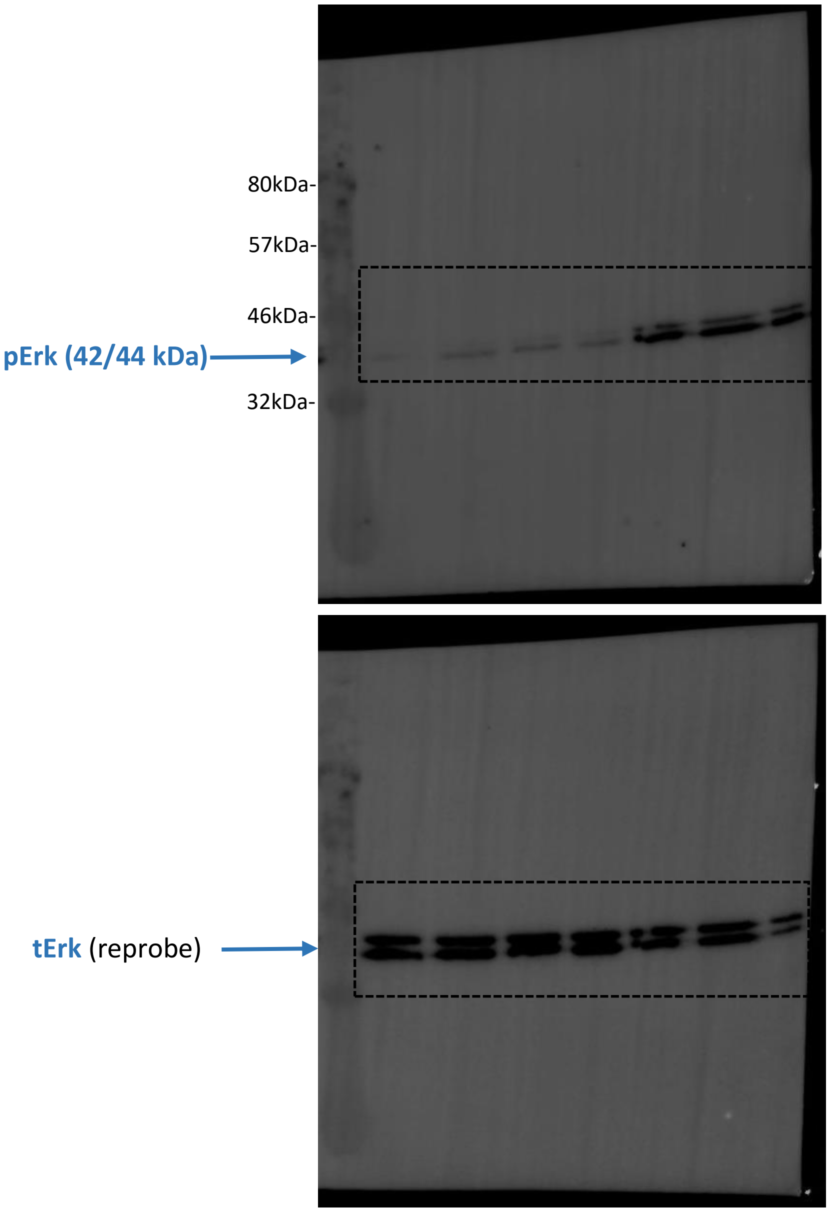

Membrane showing phospho-Erk (pErk) then stripped and reprobed for total Erk (tErk). Box with dotted line edges outlines the part of the blot displayed in the figure of the paper that includes the samples relevant to the experiment.

Figure 6b

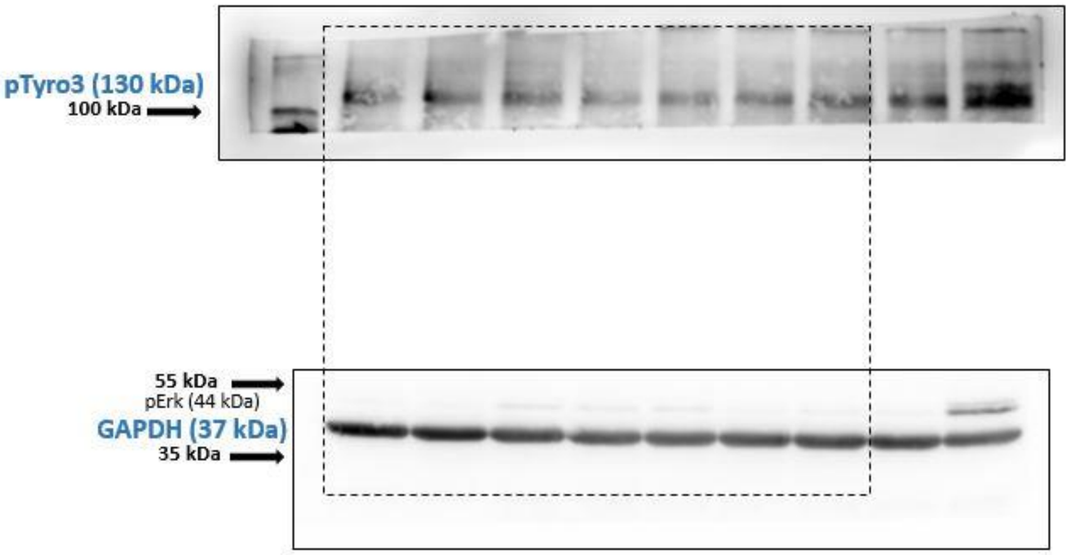

Membranes from same gel showing pTyro3 and GAPDH. Box with dotted line edges outlines the part of the blot displayed in the figure of the paper that includes the samples relevant to the experiment.

Figure 6b

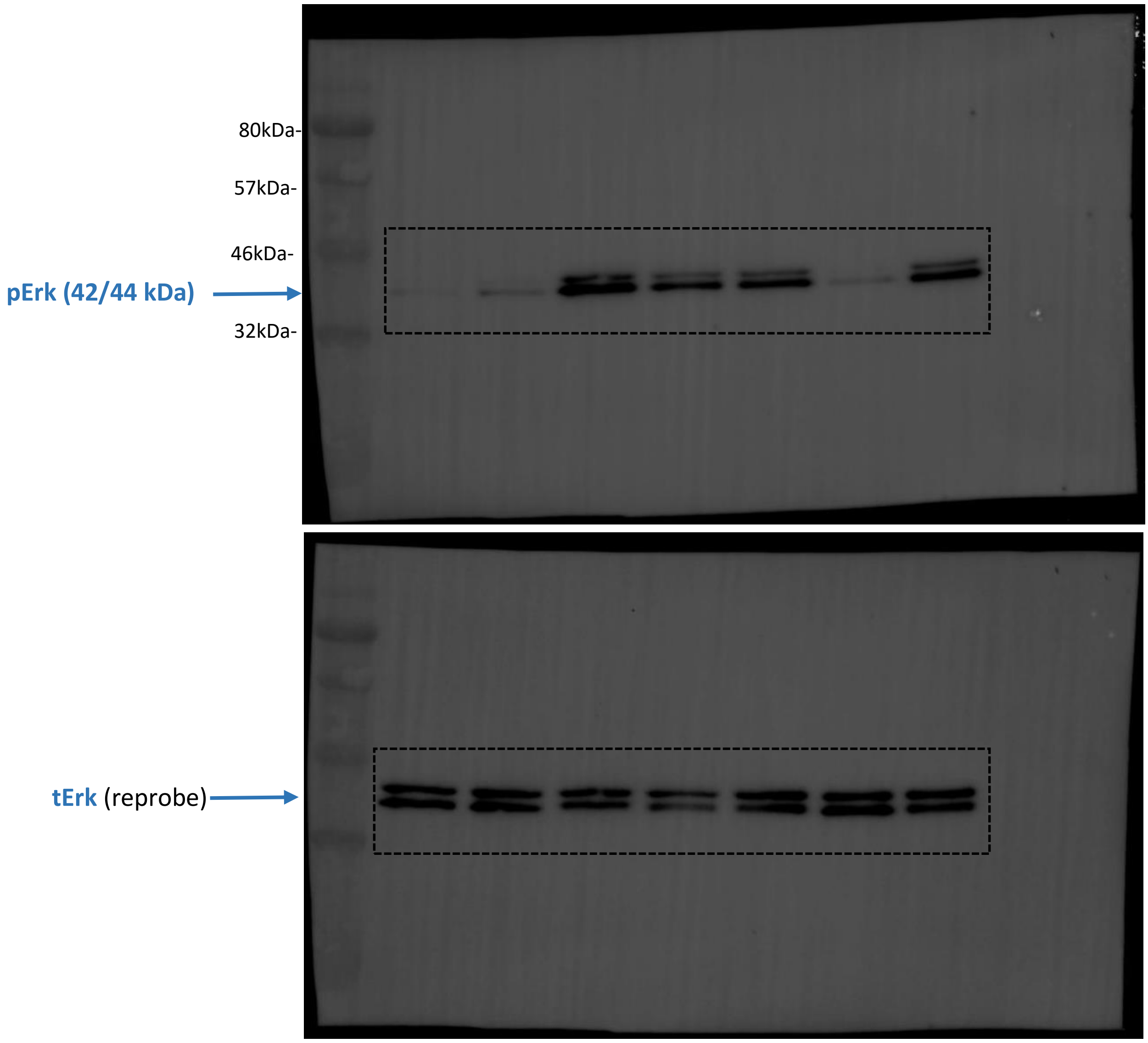

Membrane showing phospho-Erk (pErk) then stripped and reprobed for total Erk (tErk). Box with dotted line edges outlines the part of the blot displayed in the figure of the paper that includes the samples relevant to the experiment.
